# On Fourier Series of Fuzzy-Valued Functions

**DOI:** 10.1155/2014/782652

**Published:** 2014-04-10

**Authors:** Uğur Kadak, Feyzi Başar

**Affiliations:** ^1^Department of Mathematics, Faculty of Science, Bozok University, Yozgat, Turkey; ^2^Department of Mathematics, Faculty of Science, Gazi University, Ankara, Turkey; ^3^Department of Mathematics, Faculty of Arts and Sciences, Fatih University, 34500 İstanbul, Turkey

## Abstract

Fourier analysis is a powerful tool for many problems, and especially for solving various differential equations of interest in science and engineering. In the present paper since the utilization of Zadeh's Extension principle is quite difficult in practice, we prefer the idea of level sets in order to construct a fuzzy-valued function on a closed interval via related membership function. We derive uniform convergence of a fuzzy-valued function sequences and series with level sets. Also we study Hukuhara differentiation and Henstock integration of a fuzzy-valued function with some necessary inclusions. Furthermore, Fourier series of periodic fuzzy-valued functions is defined and its complex form is given via sine and cosine fuzzy coefficients with an illustrative example. Finally, by using the Dirichlet kernel and its properties, we especially examine the convergence of Fourier series of fuzzy-valued functions at each point of discontinuity, where one-sided limits exist.

## 1. Introduction

Fourier series were introduced by Joseph Fourier (1768–1830) for the purpose of solving the heat equation in a metal plate and it has long provided one of the principal methods of analysis for mathematical physics, engineering, and signal processing. While the original theory of Fourier series applies to the periodic functions occurring in wave motion, such as with light and sound, its generalizations often relate to wider settings, such as the time-frequency analysis underlying the recent theories of wavelet analysis and local trigonometric analysis. Additionally, the idea of Fourier was to model a complicated heat source as a superposition (or linear combination) of simple sine and cosine waves and to write the solution as a superposition of the corresponding eigen solutions. This superposition or linear combination is called the Fourier series.

Due to the rapid development of the fuzzy theory, however, some of these basic concepts have been modified and improved. One of them set mapping operations to the case of interval valued fuzzy sets. To accomplish this, we need to introduce the idea of the level sets of interval fuzzy sets and the related formulation of a representation of an interval valued fuzzy set in terms of its level sets. Once having these structures, we can then provide the desired extension to interval valued fuzzy sets. The effectiveness of level sets comes from not only their required memory capacity for fuzzy sets, but also from their two valued nature. This nature contributes to an effective derivation of the fuzzy-inference algorithm based on the families of the level sets. Besides, the definition of fuzzy sets by level sets offers advantages over membership functions, especially when the fuzzy sets are in universes of discourse with many elements.

Furthermore, we also study the Fourier series of periodic fuzzy-valued functions. Using a different approach, it can be shown that the Fourier series with fuzzy coefficients converges. Applying this idea, we establish some connections between the Fourier series and Fourier series of fuzzy-valued functions with the level sets. Quite recently, by using Zadeh's Extension Principle, M. Stojaković and Z. Stojaković investigated the convergence of series of fuzzy numbers in [1] and they gave some results which complete their previous results in [2]. Additionally, Talo and Başar [3] have extended the main results related to the sequence spaces and matrix transformations on the real or complex field to the fuzzy numbers with the level sets. Also, Kadak and Başar [4, 5] have recently studied the power series of fuzzy numbers and examined on some sets of fuzzy-valued sequences with the level sets and gave some properties of the level sets together with some inclusion relations in [6].

The rest of this paper is organized as follows. In Section 2, we give some required definitions and consequences related to the fuzzy numbers, sequences, and series of fuzzy numbers. We also report the most relevant and recent literature in this section. In Section 3, first, the definition of periodic fuzzy-valued function is given which will be used in the proof of our main results. In this section, Hukuhara differentiation and Henstock integration are presented according to fuzzy-valued functions which depend on *x*, *t* ∈ [*a*, *b*]. This section is terminated with the condensation of the results on uniform convergence of fuzzy-valued sequences and series. In the final section of the paper, we assert that the Fourier series of a fuzzy-valued function with 2*π* period converges and especially prove the convergence about a discontinuity point by using Dirichlet kernel and one-sided limits.

## 2. Preliminaries, Background, and Notation

A* fuzzy number* is a fuzzy set on the real axis; that is, a mapping *u* : ℝ → [0,1] which satisfies the following four conditions.

- *u* is normal; that is, there exists an *x*_0_ ∈ ℝ such that *u*(*x*_0_) = 1.
- *u* is fuzzy convex; that is, *u*[*λx* + (1 − *λ*)*y*] ≥ min⁡⁡{*u*(*x*), *u*(*y*)} for all *x*, *y* ∈ ℝ and for all *λ* ∈ [0,1].
- *u* is upper semicontinuous.
- The set ${\left[u\right]}_{0}=\bar{\left\{x\in \mathbb{R}\;:\;u(x)>0\right\}}$ is compact (cf. Zadeh [7]), where $\bar{\left\{x\in \mathbb{R}\;:\;u(x)>0\right\}}$ denotes the closure of the set {*x* ∈ ℝ : *u*(*x*) > 0} in the usual topology of ℝ.

We denote the set of all fuzzy numbers on ℝ by *E* ^1^ and called it* the space of fuzzy numbers*. *λ-level set *[*u*]_*λ*_ of *u* ∈ *E* ^1^ is defined by

(1) $$\begin{array}{c} {\left[u\right]}_{\lambda }≔\{\begin{array}{cc} \left\{t\in \mathbb{R}\;:\;u\left(t\right)\geq \lambda \right\}, & 0<\lambda \leq 1,\\ \bar{\left\{t\in \mathbb{R}\;:\;u\left(t\right)>\lambda \right\}}, & \lambda =0. \end{array} \end{array}$$

The set [*u*]_*λ*_ is closed, bounded and, nonempty interval for each *λ* ∈ [0,1] which is defined by [*u*]_*λ*_≔[*u* ^−^(*λ*), *u* ^+^(*λ*)]. ℝ can be embedded in *E* ^1^, since each *r* ∈ ℝ can be regarded as a fuzzy number $\bar{r}$ defined by

(2) $$\begin{array}{c} \bar{r}\left(x\right)≔\{\begin{array}{cc} 1, & x=r,\\ 0, & x\neq r. \end{array} \end{array}$$

**Representation Theorem** (see [8]).* Let *[*u*]_*λ*_ = [*u* ^−^(*λ*), *u* ^+^(*λ*)]* for u* ∈ *E* ^1^ *for each λ* ∈ [0,1]*. Then the following statements hold*.

- *u* ^−^ *is a bounded and nondecreasing left continuous function on *]0,1].
- *u* ^+^ *is a bounded and nonincreasing left continuous function on *]0,1].
- * The functions u* ^−^ *and u* ^+^ *are right continuous at the point λ* = 0.
- *u* ^−^(1) ≤ *u* ^+^(1).

*Conversely, if the pair of functions u* ^−^ *and u* ^+^ *satisfies the conditions (i)–(iv), then there exists a unique u* ∈ *E* ^1^ *such that *[*u*]_*λ*_≔[*u* ^−^(*λ*), *u* ^+^(*λ*)]* for each λ* ∈ [0,1]*. The fuzzy number u corresponding to the pair of functions u* ^−^ *and u* ^+^ *is defined by u* : ℝ → [0,1], *u*(*x*)≔sup⁡{*λ* : *u* ^−^(*λ*) ≤ *x* ≤ *u* ^+^(*λ*)}.

**Definition 1 ((trapezoidal fuzzy number) [9, Definition, p. 145])**

We can define trapezoidal fuzzy number *u* as *u* = (*u*_1_, *u*_2_, *u*_3_, *u*_4_); the membership function *μ*_(*u*)_ of this fuzzy number will be interpreted as follows:

(3) $$\begin{array}{c} \mu_{\left(u\right)}\left(x\right)≔\{\begin{array}{cc} \frac{x-u_{1}}{u_{2}-u_{1}}, & u_{1}\leq x\leq u_{2},\\ 1, & u_{2}\leq x\leq u_{3},\\ \frac{u_{4}-x}{u_{4}-u_{3}}, & u_{3}\leq x\leq u_{4},\\ 0, & u_{4}<x<u_{1}. \end{array} \end{array}$$

Then, the result [*u*]_*λ*_≔[*u* ^−^(*λ*), *u* ^+^(*λ*)] = [(*u*_2_ − *u*_1_)*λ* + *u*_1_, −(*u*_4_ − *u*_3_)*λ* + *u*_4_] holds for each *λ* ∈ [0,1].

Let *u*, *v*, *w* ∈ *E* ^1^ and *α* ∈ ℝ. Then the operations addition, scalar multiplication and product defined on *E* ^1^ by

(4) u⊕v=w⟺[w]λ=[u]λ⊕[v]λ ∀λ∈[0,1],⟺w−(λ)=u−(λ)+v−(λ),    w+(λ)=u+(λ)+v+(λ) ∀λ∈[0,1],    [αu]λ=α[u]λ ∀λ∈[0,1],    uv=w⟺[w]λ=[u]λ[v]λ ∀λ∈[0,1],

where it is immediate that

(5) w−(λ)=min⁡⁡{u−(λ)v−(λ),u−(λ)v+(λ),     u+(λ)v−(λ),u+(λ)v+(λ)},w+(λ)=max⁡⁡{u−(λ)v−(λ),u−(λ)v+(λ),     u+(λ)v−(λ),u+(λ)v+(λ)}

for all *λ* ∈ [0,1]. Let *W* be the set of all closed bounded intervals *A* of real numbers with endpoints $\underline{A}$ and $\bar{A}$; that is, $A≔[\underline{A},\bar{A}]$. Define the relation *d* on *W* by

(6) $$\begin{array}{c} d\left(A,B\right)≔\max\left\{\left|\underline{A}-\underline{B}\right|,\left|\bar{A}-\bar{B}\right|\right\}. \end{array}$$

Then it can easily be observed that *d* is a metric on *W* (cf. Diamond and Kloeden [10]) and (*W*, *d*) is a complete metric space (cf. Nanda [11]). Now, we can define the metric *D* on *E* ^1^ by means of the Hausdorff metric *d* as

(7) D(u,v)≔sup⁡λ∈[0,1]d([u]λ,[v]λ)≔sup⁡λ∈[0,1]max⁡⁡{|u−(λ)−v−(λ)|,         |u+(λ)−v+(λ)|}.

**Definition 2 (see [12], Definition 2.1)**

*u* ∈ *E* ^1^ is said to be a nonnegative fuzzy number if and only if *u*(*x*) = 0 for all *x* < 0. It is immediate that $u⪰\bar{0}$ if *u* is a nonnegative fuzzy number.

One can see that

(8) D(u,0¯)=sup⁡λ∈[0,1]max⁡⁡{|u−(λ)|,|u+(λ)|}=max⁡⁡{|u−(0)|,|u+(0)|}.

**Proposition 3 (see [13])**

Let *u*, *v*, *w*, *z* ∈ *E* ^1^ and *α* ∈ ℝ. Then,

- (*E* ^1^, *D*)* is a complete metric space, (cf. Puri and Ralescu [14])*.
- *D*(*αu*, *αv*) = |*α*|*D*(*u*, *v*).
- *D*(*u* ⊕ *v*, *w* ⊕ *v*) = *D*(*u*, *w*).
- *D*(*u* ⊕ *v*, *w* ⊕ *z*) ≤ *D*(*u*, *w*) + *D*(*v*, *z*).
- $\left|D\left(u,\bar{0}\right)-D\left(v,\bar{0}\right)\right|\leq D(u,v)\leq D(u,\bar{0})+D(v,\bar{0})$.

**Definition 4**

The following basic statements hold.

- [12, Definition 2.7] A sequence *u* = (*u*_*k*_) of fuzzy numbers is a function *u* from the set *ℕ* into the set *E* ^1^. The fuzzy number *u*_*k*_ denotes the value of the function at *k* ∈ *ℕ* and is called as the general term of the sequence. By *ω*(*F*), we denote the set of all sequences of fuzzy numbers.
- [12, Definition 2.9] A sequence (*u*_*n*_) ∈ *ω*(*F*) is called convergent with limit *u* ∈ *E* ^1^  if and only if for every *ε* > 0 there exists *n*_0_ = *n*_0_(*ε*) ∈ *ℕ* such that *D*(*u*_*n*_, *u*) < *ε* for all *n* ≥ *n*_0_.
- [12, Definition 2.11] A sequence (*u*_*n*_) ∈ *ω*(*F*) is called bounded if and only if the set of fuzzy numbers consisting of the terms of the sequence (*u*_*n*_) is a bounded set; that is to say, a sequence (*u*_*n*_) ∈ *ω*(*F*) is bounded if and only if there exist two fuzzy numbers *m* and *M* such that *m*⪯*u*_*n*_⪯*M* for all *n* ∈ *ℕ*. This means that *m* ^−^(*λ*) ≤ *u*_*n*_^−^(*λ*) ≤ *M* ^−^(*λ*) and *m* ^+^(*λ*) ≤ *u*_*n*_^+^(*λ*) ≤ *M* ^+^(*λ*) for all *λ* ∈ [0,1].

**Remark 5 (see [12])**

According to Definition 4, the following remarks can be given.

(a) Obviously the sequence (*u*_*n*_) ∈ *ω*(*F*) converges to a fuzzy number *u* if and only if {*u*_*n*_^−^(*λ*)} and {*u*_*n*_^+^(*λ*)} converge uniformly to *u* ^−^(*λ*) and *u* ^+^(*λ*) on [0,1], respectively.
(b) The boundedness of the sequence (*u*_*n*_) ∈ *ω*(*F*) is equivalent to the fact that
  (9) $$\begin{array}{c} \underset{n\in \mathbb{N}}{\sup}D\left(u_{n},\bar{0}\right)=\underset{n\in \mathbb{N}\;}{\sup}\underset{\lambda \in [0,1]}{\sup}\max\left\{\left|u_{n}^{-}\left(\lambda \right)\right|,\left|u_{n}^{+}\left(\lambda \right)\right|\right\}<\infty . \end{array}$$
 If the sequence (*u*_*k*_) ∈ *ω*(*F*) is bounded then the sequences of functions {*u*_*k*_^−^(*λ*)} and {*u*_*k*_^+^(*λ*)} are uniformly bounded in [0,1].

**Definition 6 (see [12])**

Let (*u*_*k*_) ∈ *ω*(*F*). Then the expression _⊕_∑_*k*_ *u*_*k*_ is called a series of fuzzy numbers with the level summation _⊕_∑. Define the sequence (*s*_*n*_) via *n*th partial level sum of the series by

(10) $$\begin{array}{c} s_{n}=u_{0}⊕u_{1}⊕u_{2}⊕\cdots ⊕u_{n} \end{array}$$

for all *n* ∈ *ℕ*. If the sequence (*s*_*n*_) converges to a fuzzy number *u* then we say that the series _⊕_∑_*k*_ *u*_*k*_ of fuzzy numbers converges to *u* and write _⊕_∑_*k*_ *u*_*k*_ which implies that

(11) $$\begin{array}{c} \underset{n\to \infty }{\lim}\overset{n}{\underset{k=0}{\sum }}u_{k}^{-}\left(\lambda \right)=u^{-}\left(\lambda \right),\;\;\underset{n\to \infty }{\lim}\overset{n}{\underset{k=0}{\sum }}u_{k}^{+}\left(\lambda \right)=u^{+}\left(\lambda \right), \end{array}$$

where the summation is in the sense of classical summation and converges uniformly in *λ* ∈ [0,1]. Conversely, if

(12) $$\begin{array}{c} \underset{k}{\sum }u_{k}^{-}\left(\lambda \right)=u^{-}\left(\lambda \right),\;\;\underset{k}{\sum }u_{k}^{+}\left(\lambda \right)=u^{+}\left(\lambda \right) \end{array}$$

converge uniformly in *λ*, then *u* = {(*u* ^−^(*λ*), *u* ^+^(*λ*)) : *λ* ∈ [0,1]} defines a fuzzy number such that *u* = _⊕_∑_*k*_ *u*_*k*_.

**Definition 7 (see [12, Definition 2.14])**

Let {*f*_*k*_(*λ*)} be a sequence of functions defined on [*a*, *b*] and *λ*_0_ ∈ ]*a*, *b*]. Then, {*f*_*k*_(*λ*)} is said to be eventually equi-left-continuous at *λ*_0_ if for any *ε* > 0 there exist *n*_0_ ∈ *ℕ* and *δ* > 0 such that |*f*_*k*_(*λ*) − *f*_*k*_(*λ*_0_)| < *ε* whenever *λ* ∈ ]*λ*_0_ − *δ*, *λ*_0_] and *k* ≥ *n*_0_. Similarly, eventually equi-right-continuity at *λ*_0_ ∈ [*a*, *b*[ of {*f*_*k*_(*λ*)} can be defined.

**Theorem 8 (see [12, Theorem 2.15])**

Let (*u*_*k*_) ∈ *ω*(*F*) such that *u*_*k*_^−^(*λ*) → *u* ^−^(*λ*) and *u*_*k*_^+^(*λ*) → *u* ^+^(*λ*), as *k* → *∞* for each *λ* ∈ [0,1]. Then, the pair of functions *u* ^−^ and *u* ^+^ determines a fuzzy number if and only if the sequences of functions {*u*_*k*_^−^(*λ*)} and {*u*_*k*_^+^(*λ*)} are eventually equi-left-continuous at each *λ*∈]0,1] and eventually equi-right-continuous at *λ* = 0.

Thus, it is deduced that the series ∑_*k*=0_^*∞*^ *u*_*k*_^−^(*λ*) = *u* ^−^(*λ*) and ∑_*k*=0_^*∞*^ *u*_*k*_^+^(*λ*) = *u* ^+^(*λ*) define a fuzzy number if the sequences

(13) $$\begin{array}{c} \left\{s_{n}^{-}\left(\lambda \right)\right\}=\left\{\overset{n}{\underset{k=0}{\sum }}u_{k}^{-}\left(\lambda \right)\right\},\;\;\left\{s_{n}^{+}\left(\lambda \right)\right\}=\left\{\overset{n}{\underset{k=0}{\sum }}u_{k}^{+}\left(\lambda \right)\right\} \end{array}$$

satisfy the conditions of Theorem 8.

**Theorem (cf. [13])**

The following statements for level addition ⊕ of fuzzy numbers and classical addition + of real scalars are valid.

- $\bar{0}$ is neutral element with respect to ⊕; that is, $u⊕\bar{0}=\bar{0}⊕u=u$ for all *u* ∈ *E* ^1^.
- With respect to $\bar{0}$, none of $u\neq \bar{r},r\in \mathbb{R}$ has opposite in *E* ^1^.
- For any *α*, *β* ∈ ℝ with *α*, *β* ≥ 0 or *α*, *β* ≤ 0, and any *u* ∈ *E* ^1^, we have (*α* + *β*)*u* = *αu* ⊕ *βu*.
- For general *α*, *β* ∈ ℝ, the above property does not hold.
- For any *α* ∈ ℝ and any *u*, *v* ∈ *E* ^1^, we have *α*(*u* ⊕ *v*) = *αu* ⊕ *αv*.
- For any *α*, *β* ∈ ℝ and any *u* ∈ *E* ^1^, we have *α*(*βu*) = (*αβ*)*u*.

### 2.1. Generalized Hukuhara Difference

Let *𝒦* be the space of nonempty compact and convex sets in the *n*-dimensional Euclidean space ℝ^*n*^. If *n* = 1, denote by *I* the set of (closed bounded) intervals of the real line. Given two elements *A*, *B* ∈ *𝒦* and *α* ∈ ℝ, the usual interval arithmetic operations, that is, addition and scalar multiplication, are defined by *A* + *B* = {*a* + *b* : *a* ∈ *A*, *b* ∈ *B*} and *αA* = {*αa* : *a* ∈ *A*}. It is well known that addition is associative and commutative and with neutral element {0}. If *α* = −1, scalar multiplication gives the opposite −*A* = (−1)*A* = {−*a* : *a* ∈ *A*} but, in general, *A* + (−*A*) ≠ 0; that is, the opposite of *A* is not the inverse of *A* in addition unless *A* is a singleton. A first consequence of this fact is that, in general, additive simplification is not valid.

To partially overcome this situation, the* Hukuhara difference*, H-difference for short, has been introduced as a set *C* for which *A* ⊖ *B*⇔*A* = *B* + *C* and an important property of ⊖ is that *A* ⊖ *A* = {0} for all *A* ∈ *𝒦* and (*A* + *B*) ⊖ *B* = *A* for all *A*, *B* ∈ *𝒦*. The H-difference is unique, but it does not always exist. A necessary condition for *A* ⊖ *B* to exist is that *A* contains a translation {*c*} + *B* of *B*.

A generalization of the Hukuhara difference proposed in [15] aims to overcome this situation.

**Definition 10 (see [15, Definition 1])**

The generalized Hukuhara difference *A* ⊖ *B* of two sets *A*, *B* ∈ *𝒦* is defined as follows:

(14) $$\begin{array}{c} A⊖B=C⟺\{\begin{array}{cc} A=B+C, & \\ B=A+\left(-1\right)C. & \end{array} \end{array}$$

**Proposition 11 (see [15])**

The following statements hold.

(a) * Let A*, *B* ∈ *𝒦 be two compact convex sets. Then, we have that*
  (i) * if the H-difference exists, it is unique and is a generalization of the usual Hukuhara difference since A* ⊖ *B* = *A* − *B, whenever A* ⊖ *B exists*.
  (ii) *A* + (−*A*) ≠ 0.
  (iii) (*A* + *B*) ⊖ *B* = *A*.
  (iv) *A* ⊖ *B* = *B* ⊖ *A* = *C*⇔*C* = {0}* and A* = *B*.
(b) * The H-difference of two intervals A* = [*a* ^−^, *a* ^+^]* and B* = [*b* ^−^, *b* ^+^]* always exists and*
  (15) $$\begin{array}{c} \left[a^{-},a^{+}\right]⊖\left[b^{-},b^{+}\right]=\left[c^{-},c^{+}\right], \end{array}$$

* where c* ^−^ = min⁡{*a* ^−^ − *b* ^−^, *a* ^+^ − *b* ^+^}, *c* ^+^ = max⁡{*a* ^−^ − *b* ^−^, *a* ^+^ − *b* ^+^}* which hold in Definition 10 are satisfied simultaneously if and only if the two intervals have the same length and* *c* ^−^ = *c* ^+^.

**Proposition 12 (see [16])**

The following statements hold.

(a) * If* *A* *and B* *are two closed intervals, then* *D*(*A*, *B*) = *D*(*A* ⊖ *B*, {0}).
(b)  * Let u* : [*a*, *b*] → *I be such that u*(*x*) = [*u* ^−^(*x*), *u* ^+^(*x*)]*. Then, we have*
  (16) $$\begin{array}{c} \underset{x\to x_{0}}{\lim}u\left(x\right)=\ell ⟺\underset{x\to x_{0}}{\lim}\left(u\left(x\right)⊖\ell \right)=\left\{0\right\},\\ \underset{x\to x_{0}}{\lim}u\left(x\right)=u\left(x_{0}\right)⟺\underset{x\to x_{0}}{\lim}\left(u\left(x\right)⊖u\left(x_{0}\right)\right)=\left\{0\right\}, \end{array}$$
  *where the limits are in the Hausdorff metric d for intervals*.

## 3. Fuzzy-Valued Functions with the Level Sets

In this chapter, we consider sequences and series of fuzzy-valued function and develop uniform convergence, Hukuhara differentiation, and Henstock integration. In addition, we present characterizations of uniform convergence signs in sequences of fuzzy-valued functions.

**Definition 13 (see [6])**

Consider a function *f* ^*t*^ from [*a*, *b*] into *E* ^1^ with respect to a membership function *μ*_*f*^*t*^_ which is called trapezoidal fuzzy number and is interpreted as follows:

(17) $$\begin{array}{c} \mu_{f^{t}}\left(x\right)≔\{\begin{array}{cc} \frac{x-f_{1}\left(t\right)}{f_{2}\left(t\right)-f_{1}\left(t\right)}, & f_{1}\left(t\right)\leq x\leq f_{2}\left(t\right),\\ 1, & f_{2}\left(t\right)\leq x\leq f_{3}\left(t\right),\\ \frac{f_{4}\left(t\right)-x}{f_{4}\left(t\right)-f_{3}\left(t\right)}, & f_{3}\left(t\right)\leq x\leq f_{4}\left(t\right),\\ 0, & f_{4}\left(t\right)<x<f_{1}\left(t\right). \end{array} \end{array}$$

Then, the membership function turns out to be *f* ^*t*^(*x*) = [*f*_*λ*_^−^(*t*), *f*_*λ*_^+^(*t*)] = [(*f*_2_(*t*) − *f*_1_(*t*))*λ* + *f*_1_(*t*), *f*_4_(*t*)−(*f*_4_(*t*) − *f*_3_(*t*))*λ*] ∈ *E* ^1^ consisting of each of the functions *f*_*λ*_^−^, *f*_*λ*_^+^ depending on *t* ∈ [*a*, *b*] for all *λ* ∈ [0,1]. Then, the function *f* ^*t*^ is said to be a fuzzy-valued function on [*a*, *b*] for all *x*, *t* ∈ [*a*, *b*].

**Remark 14**

The functions *f*_*i*_ with *i* ∈ {1,2, 3,4} given in Definition 13 are also defined for all *t* ∈ [*a*, *b*] as *f*_*i*_(*t*) = *k*, where *k* is any constant.

Now, following Kadak [17], we give the classical sets *C*_*F*_[*a*, *b*] and *B*_*F*_[*a*, *b*] consisting of the continuous and bounded fuzzy-valued functions; that is,

(18) CF[a,b]≔{ft ∣ ft:[a,b]⟶E1∋ft  continuous fuzzy-valued  function  ∀x,t∈[a,b]},BF[a,b]≔{ft ∣ ft:[a,b]⟶E1∋ft  bounded fuzzy-valued  function  ∀x,t∈[a,b]}.

Obviously, from Representation Theorem, each of the functions *f*_*λ*_^−^, *f*_*λ*_^+^ which depend on *t* ∈ [*a*, *b*] is left continuous on *λ* ∈ (0,1] and right continuous at *λ* = 0. It was shown that *C*_*F*_[*a*, *b*] and *B*_*F*_[*a*, *b*] are complete with the metric *D*_*F*_*∞*__ on *E* ^1^ defined by means of the Hausdorff metric *d* as

(19) DF∞(ft,gt)≔sup⁡x∈[a,b]{D(ft(x),gt(x))}=sup⁡x∈[a,b]{sup⁡λ∈[0,1]d([ft(x)]λ,[gt(x)]λ)}≔max⁡⁡{sup⁡λ∈[0,1] sup⁡t∈[a,b]|fλ−(t)−gλ−(t)|,      sup⁡λ∈[0,1]  sup⁡t∈[a,b]|fλ+(t)−gλ+(t)|},

where *f* ^*t*^ = *f* ^*t*^(*x*) and *g* ^*t*^ = *g* ^*t*^(*x*) are the elements of the sets *C*_*F*_[*a*, *b*] or *B*_*F*_[*a*, *b*] with *x*, *t* ∈ [*a*, *b*].

### 3.1. Generalized Hukuhara Differentiation

The concept of fuzzy differentiability comes from a generalization of the Hukuhara difference for compact convex sets. We prove several properties of the derivative of fuzzy-valued functions considered here. As a continuation of Hukuhara derivatives for real fuzzy-valued functions [18], we can define H-differentiation of a fuzzy-valued function *f* ^*t*^ with respect to level sets. For short, throughout the paper, we write *H* instead of “Hukuhara sense.”

**Definition 15**

A fuzzy-valued function *f* ^*t*^ : [*a*, *b*] → *E* ^1^ is said to be generalized H-differentiable with respect to the level sets at *x*, *t* ∈ [*a*, *b*] if

  (1) (*f* ^*t*^)′(*x*) ∈ *E* ^1^ exists such that, for all *h* > 0 sufficiently near to 0, the H-difference *f* ^*t*^(*x* + *h*) ⊖ *f* ^*t*^(*x*) exists; then the H-derivative (*f* ^*t*^)′(*x*) is given as follows:
  (20) (ft)′(x)=lim⁡h→0+[ft(x+h)⊖ft(x)h]λ=[lim⁡h→0+fλ−(t+h)−fλ−(t)h,   lim⁡h→0+fλ+(t+h)−fλ+(t)h]=[(fλ−(t))′,(fλ+(t))′],
 or (2) (*f* ^*t*^)′(*x*) ∈ *E* ^1^ exists such that, for all *h* < 0 sufficiently near to 0, the H-difference *f* ^*t*^(*x* + *h*) ⊖ *f* ^*t*^(*x*) exists; then the H-derivative (*f* ^*t*^)′(*x*) is given as follows:
  (21) (ft)′(x)=lim⁡h→0−[ft(x+h)⊖ft(x)h]λ=[lim⁡h→0−fλ−(t+h)−fλ−(t)h,   lim⁡h→0−fλ+(t+h)−fλ+(t)h]=[(fλ−)′(t),(fλ+)′(t)]

for all *x*, *t* ∈ [*a*, *b*] and *λ* ∈ [0,1].

From here, we remember that the H-derivative of *f* ^*t*^ at *x*, *t* ∈ [*a*, *b*] depends on the value *t* and the choice of a constant *λ* ∈ [0,1].

**Corollary 16**

A fuzzy-valued function *f* ^*t*^ is H-differentiable if and only if *f*_*λ*_^−^ and *f*_*λ*_^+^ are differentiable functions in the usual sense.

**Definition 17 (periodicity)**

A fuzzy-valued function *f* ^*t*^ is called periodic if there exists a constant *P* > 0 for which *f* ^*t*^(*x* + *P*) = *f* ^*t*^(*x*) for any *x*, *t* ∈ [*a*, *b*]. Thus, it can easily be seen that the conditions *f*_*λ*_^−^(*t* + *P*) = *f*_*λ*_^−^(*t*) and *f*_*λ*_^+^(*t* + *P*) = *f*_*λ*_^+^(*t*) hold for all *t* ∈ [*a*, *b*] and *λ* ∈ [0,1]. Such a constant *P* > 0 is called a period of the function *f* ^*t*^.

### 3.2. Generalized Fuzzy-Henstock Integration

**Definition 18 (see [19, Definition 8.7])**

A fuzzy valued function *f* ^*t*^ is said to be fuzzy-Henstock, in short FH-integrable, if for any *ϵ* > 0, there exists *δ* > 0 such that

(22) D(∑P(v−u)ft(ξ),I)  =sup⁡λ∈[0,1]max⁡⁡{|∑P(v−u)fλ−(t)−Iλ−|,          |∑P(v−u)fλ+(t)−Iλ+|}<ϵ

for any division *P* = {[*u*, *v*]; *ξ*} of [*a*, *b*] with the norms Δ(*P*) < *δ*, where *I*≔(FH)∫_*a*_^*b*^ *f* ^*t*^(*x*)*dx* and *t* ∈ [*a*, *b*], and *f* ^*t*^ is FH-integrable. One can conclude that ∑_*P*_ in (22) denotes the usual Riemann sum for any division *P* of [*a*, *b*].

**Theorem 19 (see [19, Theorem 8.8])**

Let *f* ^*t*^ ∈ *C*_*F*_[*a*, *b*] and FH-integrable on [*a*, *b*]. If there exists *x*_0_ ∈ [*a*, *b*] such that *f*_*λ*_^−^(*x*_0_) = *f*_*λ*_^+^(*x*_0_) = 1, then

(23) $$\begin{array}{c} {\left[(\text{FH})\overset{b}{\underset{a}{\int }}f^{t}(x)dx\right]}_{\lambda }=\left[\overset{x_{0}}{\underset{a}{\int }}f_{\lambda }^{-}\left(t\right)dt,\overset{b}{\underset{x_{0}}{\int }}f_{\lambda }^{+}\left(t\right)dt\right]. \end{array}$$

**Remark 20**

We remark that the integrals ∫_*a*_^*b*^ *f*_*λ*_^±^(*t*)*dt* in (23) exist in the usual sense for all *λ* ∈ [0,1] and *t* ∈ [*a*, *b*]. It is easy to see that the pair of functions *f*_*λ*_^±^ : [*a*, *b*] → ℝ are continuous.

**Remark 21**

Note that if *f* ^*t*^ is periodic fuzzy-valued function and FH-integrable on any interval of length *P*, then it is FH-integrable on any other of the same length, and the value of the integral is the same; that is,

(24) $$\begin{array}{c} {\left[(\text{FH})\overset{a+P}{\underset{a}{\int }}f^{t}(x)dx\right]}_{\lambda }={\left[(\text{FH})\overset{b+P}{\underset{b}{\int }}f^{t}(x)dx\right]}_{\lambda } \end{array}$$

for all *x*, *t* ∈ [*a*, *b*] and *λ* ∈ [0,1].

This property is an immediate consequence of the interpretation of an integral as an area. In fact, each integral (24) equals the area bounded by the curves *f* ^±^(*t*), the straight lines *x* = *a* and *x* = *b*, and the closed interval [*a*, *b*] of *x*-axis. In the present case, the areas represented by two integrals are the same because of the periodicity of *f* ^*t*^. Hereafter, when we say that a fuzzy-valued function *f* ^*t*^ with period *P* is FH-integrable, we mean that it is FH-integrable on an interval of length *P*. It follows from the property just proved that *f* ^*t*^ is also FH-integrable on any interval of finite length.

**Definition 22 (see [6] (uniform convergence))**

Let {*f*_*n*_^*t*^(*x*)} be a sequence of fuzzy-valued functions defined on a set *A*⊆ℝ. We say that {*f*_*n*_^*t*^(*x*)} converges pointwise on *A* if for each *x* ∈ *A* the sequence {*f*_*n*_^*t*^(*x*)} converges for all *x*, *t* ∈ *A* and *λ* ∈ [0,1]. If a sequence {*f*_*n*_^*t*^(*x*)} converges pointwise on a set *A*, then we can define *f* ^*t*^ : *A* → *E* ^1^ by

(25) $$\begin{array}{c} \underset{n\to \infty }{\lim}f_{n}^{t}\left(x\right)=f^{t}\left(x\right)\;\text{for}\;\text{each}\;x,t\in A. \end{array}$$

In other words, {*f*_*n*_^*t*^(*x*)} converges to *f* ^*t*^ on *A* if and only if for each *x* ∈ *A* and for an arbitrary *ϵ* > 0, there exists an integer *N* = *N*(*ϵ*, *x*) such that *D*(*f*_*n*_^*t*^(*x*), *f* ^*t*^(*x*)) < *ϵ* whenever *n* > *N*. The integer *N* in the definition of pointwise convergence may, in general, depend on both *ϵ* > 0 and *x* ∈ *A*. If, however, one integer can be found that works for all points in *A*, then the convergence is said to be uniform. That is, a sequence of fuzzy-valued functions {*f*_*n*_^*t*^(*x*)} converges uniformly to *f* ^*t*^ on a set *A* if, for each *ϵ* > 0, there exists an integer *N*(*ϵ*) such that

(26) $$\begin{array}{c} D\left(f_{n}^{t}\left(x\right),f^{t}\left(x\right)\right)<\epsilon \;\text{whenever}\;n>N\left(\epsilon \right),\;\;\forall x,t\in A. \end{array}$$

Obviously, the sequence (*f*_*n*_^*t*^) of fuzzy-valued functions converges to a fuzzy valued-function *f* ^*t*^ if and only if {(*f*_*λ*_^−^)_*n*_(*t*)} and {(*f*_*λ*_^+^)_*n*_(*t*)} converge uniformly to *f*_*λ*_^−^(*t*) and *f*_*λ*_^+^(*t*) in *λ* ∈ [0,1], respectively. Often, we say that *f* ^*t*^ is the uniform limit of the sequence {*f*_*n*_^*t*^(*x*)} on *A* and write *f*_*n*_^*t*^ → *f* ^*t*^, *n* → *∞*, uniformly on *A*.

Now, as a consequence of Definition 22, the following theorem determines the characterization of uniform convergence of fuzzy-valued sequences.

**Theorem 23 (see [6])**

Let *x*, *t* ∈ *A* and *λ* ∈ [0,1]. Then, the following statements are valid.

(i) A sequence of fuzzy-valued functions {*f*_*n*_^*t*^(*x*)} defined on a set *A*⊆ℝ converges uniformly to a fuzzy-valued function *f* ^*t*^ on *A* if and only if
  (27) δn=sup⁡x∈[a,b]D(fnt(x),ft(x))=sup⁡x∈[a,b]{sup⁡λ∈[0,1]d([fnt(x)]λ,[ft(x)]λ)}             with  lim⁡n→∞δn=0¯.
(ii) The limit of a uniformly convergent sequence of continuous fuzzy-valued functions {*f*_*n*_^*t*^} on a set *A* is continuous. That is, for each *a* ∈ *A*,
  (28) $$\begin{array}{c} \underset{x\to a}{\lim}\left[\underset{n\to \infty }{\lim}f_{n}^{t}\left(x\right)\right]=\underset{n\to \infty }{\lim}\left[\underset{x\to a}{\lim}f_{n}^{t}\left(x\right)\right]. \end{array}$$

**Theorem 24 (interchange of limit and integration)**

Suppose that *f*_*n*_^*t*^(*x*) ∈ *C*_*F*_[*a*, *b*] for all *n* ∈ *ℕ* such that {*f*_*n*_^*t*^(*x*)} converges uniformly to *f* ^*t*^(*x*) on [*a*, *b*]. By combining this and the inclusion (28), the equalities

(29) lim⁡n→∞[(FH)∫abfnt(x)dx]λ=[(FH)∫ablim⁡n→∞fnt(x)dx]λ=[(FH)∫abft(x)dx]λ

hold, where the integral (FH)∫_*a*_^*b*^ *f* ^*t*^(*x*)*dx* exists for all *x*, *t* ∈ [*a*, *b*] and *λ* ∈ [0,1]. Also, for each *p* ∈ [*a*, *b*], it is trivial that

(30) lim⁡n→∞[(FH)∫apfnt(x)dx]λ=[(FH)∫apft(x)dx]λ=[∫apfλ−(t)dt,∫apfλ+(t)dt]

and the convergence is uniform on [*a*, *b*].

**Proof**

Note that by Part (ii) of Theorem 23, *f* ^*t*^ is continuous on [*a*, *b*], so that (FH)∫_*a*_^*b*^ *f* ^*t*^(*x*)*dx* exists. Let *ε* > 0 be given. Then, since *f*_*n*_^*t*^ → *f* ^*t*^ uniformly on [*a*, *b*], there is an integer *N* = *N*(*ε*) such that

(31) D[fnt(x),ft(x)]  =max⁡⁡{sup⁡λ∈[0,1] sup⁡t∈[a,b]|fn(t)λ−−fλ−(t)|,       sup⁡λ∈[0,1] sup⁡t∈[a,b]|fn(t)λ+−fλ+(t)|}<ε(b−a)

for *n* > *N*(*ε*). Again, since the distance function *D*(*f*_*n*_^*t*^, *f* ^*t*^) is continuous on [*a*, *b*], it follows

(32) D[(FH)∫abfnt(x)dx,(FH)∫abft(x)dx]  =sup⁡λ∈[0,1]d([(FH)∫abfnt(x)dx]λ,         [(FH)∫abft(x)dx]λ)

and the equality on rigt-hand side in (32) is evaluated as

(33) sup⁡λ∈[0,1]max⁡{|∫ab[fn(t)λ−−f(t)λ−]dt|,        |∫ab[fn(t)λ+−f(t)λ+]dt|}  ≤∫abmax⁡{sup⁡λ∈[0,1] sup⁡t∈[a,b]|fn(t)λ−−f(t)λ−|,         sup⁡λ∈[0,1] sup⁡t∈[a,b]|fn(t)λ+−f(t)λ+|}dt  <εb−a(b−a)=ε

for *n* > *N*(*ε*). Since *ε* is arbitrary, this step completes the proof.

The hypothesis of Theorem 24 is sufficient for our purposes and may be used to show the nonuniform convergence of the sequence {*f*_*n*_^*t*^(*x*)} on [*a*, *b*]. Also, it is important to point out that a direct analogue of Theorem 24 for H-derivatives is not true.

**Remark 25**

The uniform convergence of {*f*_*n*_^*t*^(*x*)} is sufficient but is not necessary. In other words the conclusion of Theorem 24 holds without {*f*_*n*_^*t*^(*x*)} being convergent uniformly on [*a*, *b*].

**Definition 26**

The series _⊕_∑_*k*=1_^*∞*^ *f*_*k*_^*t*^(*x*) is said to be uniformly convergent to a fuzzy-valued function *f* ^*t*^(*x*) on *A* if the partial level sum {*S*_*n*_^*t*^(*x*)} converges uniformly to *f* ^*t*^(*x*) on *A*. That is, the series converges uniformly to *f* ^*t*^(*x*) if, given any *ε* > 0, there exists an integer *n*_0_(*ε*) such that

(34) D[∑k=1∞fkt(x),ft(x)⊕]  =max⁡⁡{sup⁡λ∈[0,1] sup⁡t∈[a,b]|∑k=1∞fk(t)λ−−f(t)λ−|,       sup⁡λ∈[0,1] sup⁡t∈[a,b]|∑k=1∞fk(t)λ+−f(t)λ+|}<ε

for all *x*, *t* ∈ *A* and *λ* ∈ [0,1] whenever *n* ≥ *n*_0_(*ε*).

**Corollary**

If {*f*_*k*_^*t*^(*x*)} is a continuous fuzzy-valued function on *A*⊆ℝ for each *k* ≥ 1 and _⊕_∑_*k*≥1_ *f*_*k*_^*t*^(*x*) is uniformly convergent to *f* ^*t*^(*x*) on *A*, then *f* ^*t*^ is continuous on *A* for all *x*, *t* ∈ *A*.

**Corollary 28 (interchange of summation and integration)**

Suppose that {*f*_*k*_^*t*^(*x*)} is a sequence in *C*_*F*_[*a*, *b*] and _⊕_∑_*k*=0_^*∞*^ *f*_*k*_^*t*^(*x*) converges uniformly to *f* ^*t*^(*x*) on [*a*, *b*]. Then,

(35) [(FH)⊕∑k=0∞∫abfkt(x)dx]λ=[(FH)∫ab⊕∑k=0∞ft(x)dx]λ=[(FH)∫abfkt(x)dx]λ,

where (FH)∫_*a*_^*b*^ *f* ^*t*^(*x*)*dx* exists for all *x*, *t* ∈ [*a*, *b*] and *λ* ∈ [0,1].

Now, we give an important trigonometric system whose special case of one of the systems of functions is applying to the well-known inequalities.

By a trigonometric system we mean the system of 2*π* periodic *cosine* and *sine* functions which is given by

(36) 1,cos⁡⁡(x),sin⁡(x),cos⁡⁡(2x),sin⁡(2x),…,cos⁡⁡(nx),sin⁡(nx),…,

for all *n* ∈ *ℕ*. We now prove some auxiliary formulas for any positive integer *n* such that ∫_−*π*_^*π*^cos⁡⁡(*nx*)*dx* = ∫_−*π*_^*π*^sin⁡(*nx*)*dx* = 0. Therefore, one can see by using trigonometric identities that

(37) ∫−ππcos⁡⁡mxcos⁡⁡nx dx={0,m≠n,2π,m=n=0,π,m=n≠0,∫−ππsin⁡mxsin⁡nx dx={0,m≠n,π,m=n≠0.

It is known that the integral of a periodic function is the same over any interval whose length equals its period. Therefore, the formulas are valid not only for the interval [−*π*, *π*] but also for any interval [*a*, *a* + 2*π*]; that is, the system (36) is orthogonal on every such interval, where *a* ∈ ℝ.

## 4. Fourier Series for Fuzzy-Valued Functions of Period 2*π*

**Definition 29**

Let *f* ^*t*^ be a 2*π*-periodic fuzzy-valued function on a set *A*. The Fourier series of fuzzy-valued function *f* ^*t*^ of period 2*π* is defined as follows:

(38) ft(x)≅a0⊕⊕∑n=1∞(ancos⁡⁡nx⊕bnsin⁡nx)

with respect to the fuzzy coefficients *a*_*n*_ and *b*_*n*_, which converges uniformly in *λ* ∈ [0,1] for all *n* ∈ *ℕ* and *x*, *t* ∈ *A*.

Now, we can calculate the Fourier coefficients *a*_0_, *a*_*n*_, and *b*_*n*_ with respect to the level sets; that is, *a*_*n*_ = [(*a*_*n*_)_*λ*_^−^, (*a*_*n*_)_*λ*_^+^]. We derive from (38) by FH-integrating over [−*π*, *π*] that

(39) [(FH)∫−ππft(x)dx]λ =[(FH)∫−ππa0dx]λ  ⊕⊕∑n=1∞[(FH)∫−ππ(ancos⁡⁡nx⊕bnsin⁡nx)dx]λ.

As an extension of the relation (39) to write with level sets, we have

(40) [∫−ππfλ−(t)dt,∫−ππfλ+(t)dt] =[∫−ππ(a0)λ−(t)dt,∫−ππ(a0)λ+(t)dt]  ⊕∑n=1∞[∫−ππ(an)λ−(t)cos⁡⁡nt dt,∫−ππ(an)λ+(t)cos⁡⁡nt dt]  ⊕∑n=1∞[∫−ππ(bn)λ−(t)sin⁡nt dt,∫−ππ(bn)λ+(t)sin⁡nt dt]

for each *λ* ∈ [0,1] and *x*, *t* ∈ [*a*, *b*]. By taking into account the formulas of orthogonal system in (36) for each *m*, *n* ∈ *ℕ* with *m* ≠ *n*, to get *a*_*n*_, and by multiplying (39) by cos⁡⁡*mx*, we obtain by FH-integrating it over [−*π*, *π*] that

(41) [(FH)∫−ππft(x)dx]λ  =[(FH)∫−ππa0dx]λ   ⊕⊕∑n=1∞[(FH)∫−ππ{ancos⁡⁡mxcos⁡⁡nx             ⊕bnsin⁡nxcos⁡⁡mx}dx]λ.

Similarly to get *b*_*n*_, multiplying (39) by sin⁡*mx* and we present by FH-integrating it over [−*π*, *π*] that the coefficients *a*_0_, *a*_*n*_, and *b*_*n*_ with respect to the level sets are derived that

(42) an=1π[(FH)∫−ππft(x)cos⁡⁡nx dx]λ=1π[∫−ππfλ−(t)cos⁡⁡nt dt,∫−ππfλ+(t)cos⁡⁡nt dt],                      (n≥0), bn=1π[(FH)∫−ππft(x)sin⁡nx dx]λ=1π[∫−ππfλ−(t)sin⁡nt dt,∫−ππfλ+(t)sin⁡nt dt],                     (n≥1).

Combining the trigonometric identity cos⁡⁡(*a* − *b*) = cos⁡⁡*a*cos⁡⁡*b* + sin⁡*a*sin⁡*b* with *a* = *ns* and *b* = *nx* and substituting the formulas (42) in (38), one can observe that

(43) ft(x)≅12π[(FH)∫−ππft(x)dx]λ ⊕⊕∑n=1∞1π[(FH)∫−ππft(x)cos⁡⁡(ns−nx)dx]λ

which is the desired alternate form of the Fourier series of fuzzy-valued function *f* ^*t*^ on the interval [−*π*, *π*] for each *λ* ∈ [0,1].

Therefore, in looking for a trigonometric series of fuzzy-valued functions whose level sum is a given fuzzy-valued function *f* ^*t*^, it is natural to examine first the series whose coefficients are given by (42). The trigonometric series with these coefficients is called the Fourier series of fuzzy-valued function *f* ^*t*^. Incidentally, we note that fuzzy coefficients involve FH-integrating of a fuzzy-valued function of period 2*π*. Therefore, the interval of integration can be replaced by any other interval of length 2*π*.

**Remark 30**

Let *f* ^*t*^ be any fuzzy-valued function defined only on [−*π*, *π*] in trigonometric series. In this case, nothing at all is said about the periodicity of *f* ^*t*^. In fact, if the Fourier series of fuzzy-valued functions turns out to converge to *f* ^*t*^, then, since it is a periodic function, the level sum of this automatically gives us the required periodic extension of *f* ^*t*^.

**Example 31**

Let *f* ^*t*^ be 2*π*-periodic fuzzy valued function and FH-integrable on [−*π*, *π*] with trapezoidal form defined by

(44) $$\begin{array}{c} f^{t}\left(x\right)≔\{\begin{array}{cc} \frac{x+\pi }{t+\pi }, & -\pi \leq x\leq t,\\ 1, & t\leq x\leq \pi -t,\\ \frac{\pi -x}{t} & \pi -t\leq x\leq \pi ,\\ 0, & x<-\pi ,\;\;x>\pi \end{array} \end{array}$$

which is FH-integrable on [−*π*, *π*] for each *x*, *t* ∈ [*a*, *b*] and *λ* ∈ [0,1]. By using Definition 1, the level set [*f* ^*t*^]_*λ*_ of the membership function *f* ^*t*^ can be written as follows:

(45) $$\begin{array}{c} {\left[f^{t}\right]}_{\lambda }≔\left[f_{\lambda }^{-}\left(t\right),f_{\lambda }^{+}\left(t\right)\right]=\left[t\lambda +\pi \left(\lambda -1\right),\pi -t\lambda \right]. \end{array}$$

Therefore, we calculate the fuzzy Fourier coefficients *a*_0_, *a*_*n*_, and *b*_*n*_ as follows:

(46) a0=12π[∫−ππ[tλ+π(λ−1)]dt,∫−ππ[π−tλ]dt]=[π(λ−1),π],an=1π[∫−ππ[tλ+π(λ−1)]cos⁡⁡nt dt,∫−ππ[π−tλ]cos⁡⁡nt dt]=[0,0]=[0]λ,bn=1π[∫−ππ[tλ+π(λ−1)]sin⁡nt dt,∫−ππ[π−tλ]sin⁡nt dt]=(−1)n[−2λn,2λn].

By considering above coefficients in (38) and the condition *k*[*u*_*λ*_^−^, *u*_*λ*_^+^] = [*ku*_*λ*_^+^, *ku*_*λ*_^−^] if *k* < 0, we have

(47) ft(x)≅[π(λ−1),π] ⊕⊕∑n=1∞(−1)n[−2λn,2λn]sin⁡nx=[π(λ−1)+2λsin⁡x−λsin⁡2x  +2λ3sin⁡3x−⋯,π−2λsin⁡x  +λsin⁡2x−2λ3sin⁡3x+⋯].

**Definition 32 (complex form)**

Let *f* ^*t*^ be a fuzzy-valued function and FH-integrable on [−*π*, *π*], and its Fourier series is in the form (38). By substituting Euler's well-known formulas related to the trigonometric and exponential functions: *e* ^*ix*^ = cos⁡⁡*x* + *i*sin⁡*x* and cos⁡⁡*nx* = (*e* ^*inx*^ + *e* ^−*inx*^)/2, sin⁡*nx* = (*e* ^*inx*^ − *e* ^−*inx*^)/2*i* in (38), the complex form of Fourier series of fuzzy-valued function *f* ^*t*^ is given by

(48) ft(x)≅12a0⊕⊕∑n=1∞[12(an⊕ibn)einx⊕12(an⊖ibn)e−inx],

where the H-difference (*a*_*n*_ ⊖ *ib*_*n*_) exists for all *n* ∈ *ℕ* and *x*, *t* ∈ *A*.

If we set

(49) $$\begin{array}{c} c_{0}=\frac{1}{2}a_{0},\;\;c_{n}=\frac{1}{2}\left(a_{n}⊕ib_{n}\right),\\ c_{-n}=\frac{1}{2}\left(a_{n}⊖ib_{n}\right), \end{array}$$

and then the *M*th partial sum of the series (48) and hence of the series (38), can be written in the form

(50) stM(x)=c0⊕⊕∑n=1M(cneinx⊕c−ne−inx).

Therefore, it is natural to write

(51) ft(x)≅⊕∑n=−∞∞cneinx.

The coefficients *c*_*n*_ are given by (49) called the complex Fourier fuzzy coefficients and satisfy the following relation:

(52) $$\begin{array}{c} c_{n}=\frac{1}{2\pi }{\left[\left(\text{FH}\right)\overset{\pi }{\underset{-\pi }{\int }}f^{t}\left(x\right)e^{-inx}dx\right]}_{\lambda }. \end{array}$$

**Definition 33**

Let *f* ^*t*^ be any fuzzy-valued function on [*a*, *b*], defined either on the whole *x*-axis or on some intervals. Then, *f* ^*t*^ is said to be an even function if *f* ^*t*^(−*x*) = *f*(*x*) for every *x*. Thus, the conditions *f*_*λ*_^−^(−*t*) = *f*_*λ*_^−^(*t*) and *f*_*λ*_^+^(−*t*) = *f*_*λ*_^+^(*t*) hold for all *t* ∈ [*a*, *b*] and *λ* ∈ [0,1].

**Definition 34**

Let *f* ^*t*^ be an even function on [−*π*, *π*], or else an even periodic function. Then, the Fourier fuzzy coefficients of *f* ^*t*^ are

(53) an=1π[(FH)∫−ππft(x)cos⁡⁡nx dx]λ=2π[(FH)∫0πft(x)cos⁡⁡nx dx]λ

and *b*_*n*_ = [0]_*λ*_. Therefore, Fourier series of an *f* ^*t*^ consists of cosines; that is,

(54) ft(x)≅a0⊕⊕∑n=1∞ancos⁡⁡nx.

**Remark 35**

By taking into account Definition 13, one can conclude that a fuzzy valued function can not be odd. Because the functions *f* ^−^ and *f* ^+^ that are given in Representation Theorem can not be odd functions. Therefore, the Fourier series of fuzzy valued function do not consist of the sines. However, we can define the sines without using the oddness property as follows.

**Definition 36**

Let *f* ^*t*^ be a periodic fuzzy-valued function on an closed interval. Then, if the fuzzy Fourier coefficient $a_{n}=\bar{0}$, then fuzzy Fourier series consists of sines, that is,

(55) ft(x)≅⊕∑n=1∞bnsin⁡nx.

**Definition 37 (one-sided H-derivatives)**

Let *f* ^*t*^ be any fuzzy-valued function on *A* and continuous except possibly for a finite number of finite jumps. This means that *f* ^*t*^ is permitted to be discontinuous at a finite number of points in each period, but at these points we assume that both of the one-sided limits exist and are finite. For convenience, we introduce this notation for these limits,

(56) ft(x0−)=[lim⁡t→t0−0fλ−(t),lim⁡t→t0−0fλ+(t)]=[fλ−(t0−),fλ+(t0−)]=lim⁡x→x0−0ft(x),ft(x0+)=[lim⁡t→t0+0fλ−(t),lim⁡t→t0+0fλ+(t)]=[fλ−(t0+),fλ+(t0+)]=lim⁡x→x0+0ft(x)

for all *x*, *t* ∈ *A*. In addition, we suppose that the generalized left-hand H-derivative (*f*_*L*_^*t*^)′(*x*_0_) exists and is defined by

(57) (fLt)′(x0)=lim⁡h→0[ft(x0+h)⊖ft(x0−)h]λ=lim⁡u→0[ft(x0−u)⊖ft(x0−)−u]λ.

Thus, we can write

(58) (fLt)′(x0)=[lim⁡h→0−fλ−(t0+h)−fλ−(t0−)h,   lim⁡h→0−fλ+(t0+h)−fλ+(t0−)h]=[(fλ−)′(t0),(fλ+)′(t0)].

If *f* ^*t*^ is continuous at *x*_0_, this coincides with the usual left-hand derivative; if *f* ^*t*^ has a discontinuity at *x*_0_, we take care to use the left-hand instead of just writing *f* ^*t*^(*x*_0_).

Symmetrically, we shall also assume that the generalized right-hand H-derivative (*f*_*R*_^*t*^)′(*x*_0_) exists and is defined by

(59) (fRt)′(x0)=lim⁡h→0[ft(x0+h)⊖ft(x0+)h]λ=[lim⁡h→0+fλ−(t0+h)−fλ−(t0+)h,   lim⁡h→0+fλ+(t0+h)−fλ+(t0+)h].

We begin with quoting the following lemmas which are needed in proving the convergence of a Fourier series of fuzzy-valued functions at each point of discontinuity.

**Lemma 38 (see [20, Lemma 2.11.3] (Dirichlet kernel))**

The Dirichlet kernel *D*_*N*_ is defined by

(60) $$\begin{array}{c} D_{N}\left(u\right)=\frac{1}{2}+\overset{N}{\underset{n=1}{\sum }}\cos nu, \end{array}$$

where *n* is a positive integer. The Dirichlet kernel *D*_*N*_ has the following two properties. The first involves the definite integral of *D*_*N*_(*u*) on the interval [0, *π*]. That is,

(61) $$\begin{array}{c} \overset{\pi }{\underset{0}{\int }}D_{N}\left(u\right)du=\overset{\pi }{\underset{0}{\int }}\left[\frac{1}{2}+\overset{N}{\underset{n=1}{\sum }}\cos nu\right]du=\frac{\pi }{2} \end{array}$$

and the second property is

(62) $$\begin{array}{c} D_{N}\left(u\right)=\frac{\sin\left(\left(2N+1\right)u/2\right)}{2\sin\left(u/2\right)}. \end{array}$$

**Lemma 39**

Let *g* ^*t*^ ∈ *C*_*F*_[0, *π*] and FH-integrable on [0, *π*[; then

(63) $$\begin{array}{c} \underset{n\to \infty }{\lim}{\left[(\text{FH})\overset{\pi }{\underset{0}{\int }}g^{t}\left(u\right)\sin\left(nu+\frac{u}{2}\right)du\right]}_{\lambda }={\left[0\right]}_{\lambda }, \end{array}$$

where *n* is a positive integer.

**Proof**

By taking into account FH-integration and the Dirichlet kernel defined in Lemma 38, the integral in (63) can be evaluated as

(64) [∫0πgλ−(t)[sin⁡(t2)cos⁡⁡nt+cos⁡⁡(t2)sin⁡nt]dt, ∫0πgλ+(t)[sin(t2)cos⁡⁡nt+cos⁡⁡(t2)sin⁡nt]dt]  =π2[(an)λ−+(bn)λ−,(an)λ++(bn)λ+]  =π2an⊕π2bn,

where (*a*_*n*_)_*λ*_^−^ and (*a*_*n*_)_*λ*_^+^ are the Fourier cosine coefficients of *g*_*λ*_^−^(*t*)sin⁡(*t*/2) and *g*_*λ*_^+^(*t*)sin⁡(*t*/2) on the interval ]0, *π*[ in Definition 34. Similarly, (*b*_*n*_)_*λ*_^−^ and (*b*_*n*_)_*λ*_^+^ are the Fourier sine coefficients of *g*_*λ*_^−^(*t*)cos⁡⁡(*t*/2) and *g*_*λ*_^+^(*t*)cos⁡⁡(*t*/2) on the interval ]0, *π*[ in Definition 36, respectively. Taking the limit on both sides and using orthogonal formulas, we have $\lim_{n\to \infty }a_{n}=\bar{0}$ and $\lim_{n\to \infty }b_{n}=\bar{0}$; then we have

(65) lim⁡n→∞[(FH)∫0πgt(u)sin⁡(nu+u2)du]λ  =lim⁡n→∞(π2an⊕π2bn)=[0]λ

for all *u*, *t* ∈ [0, *π*].

**Lemma 40**

Suppose that *g* ^*t*^ ∈ *C*_*F*_[0, *π*] and (*g*_*R*_^*t*^)′(0) exists. Then,

(66) $$\begin{array}{c} \underset{N\to \infty }{\lim}{\left[(\text{FH})\overset{\pi }{\underset{0}{\int }}g^{t}(u)D_{N}(u)du\right]}_{\lambda }=\frac{\pi }{2}g^{t}\left(0+\right). \end{array}$$

**Proof**

Let *g* ^*t*^ ∈ *C*_*F*_[0, *π*] and let (*g*_*R*_^*t*^)′(0) exist. Then, we have from (66) that

(67) [(FH)∫0πgt(u)DN(u)du]λ =[∫0π[gλ−(t)−gλ−(0+)+gλ−(0+)]DN(t)dt,   ∫0π[gλ+(t)−gλ+(0+)+gλ+(0+)]DN(t)dt]

and this equality turns out to be

(68) [∫0π[gλ−(t)−gλ−(0+)]DN(t)dt +∫0πgλ−(0+)DN(t)dt, ∫0π[gλ+(t)−gλ+(0+)]DN(t)dt +∫0πgλ+(0+)DN(t)dt]

for all *t* ∈ [0, *π*] and *λ* ∈ [0,1]. Each of the integrals on the right-hand side will be considered individually. First, using the second property of the Dirichlet kernel in (62), we get

(69) ∫0π[gλ±(t)−gλ±(0+)]DN(t)dt =∫0π[gλ±(t)−gλ±(0+)]sin⁡(nt+(t/2))2sin⁡(t/2)dt =∫0πgλ±(t)−gλ±(0+)2(t/2)t/2sin⁡(t/2)sin⁡(nt+t2)dt =∫0πgλ±(t)−gλ±(0+)t−0t/2sin⁡(t/2)sin⁡(nt+t2)dt.

Let *h* ^*t*^ be a fuzzy-valued function defined by *h* ^*t*^(*u*) = [*g*_*λ*_^±^(*t*) − *g*_*λ*_^±^(0+)]*t*/[2(*t* − 0)sin(*t*/2)] and continuous on ]0, *π*]. For the sake of argument, it must be established that *h*_*λ*_^±^(*t*) is piecewise continuous on (0, *π*). The piecewise continuity of *h*_*λ*_^±^(*t*) hinges on the right-side limit at *t* = 0.

Consider

(70) $$\begin{array}{c} \underset{t\to 0^{+}}{\lim}h^{t}\left(u\right)=\underset{t\to 0^{+}}{\lim}\frac{g_{\lambda }^{\pm }\left(t\right)-g_{\lambda }^{\pm }\left(0+\right)}{t-0}\frac{t/2}{\sin\left(t/2\right)}. \end{array}$$

Provided that the individual limits at (68) exist. The continuity of *h* ^*t*^ allows the application of Lemma 39, so that

(71) lim⁡N→∞∫0π[gλ±(t)−gλ±(0+)]DN(t)dt  =lim⁡N→∞[ht(u)sin⁡(nu+u2)du]λ=[0]λ.

As for the second integral on (68), it follows that

(72) $$\begin{array}{c} \underset{N\to \infty }{\lim}\overset{\pi }{\underset{0}{\int }}g_{\lambda }^{\pm }\left(0+\right)D_{N}\left(t\right)dt=\frac{\pi }{2}g_{\lambda }^{\pm }\left(0+\right). \end{array}$$

Combining the results, it follows that

(73) lim⁡N→∞[(FH)∫0πgt(u)DN(u)du]λ  =[lim⁡N→∞∫0π[gλ−(t)−gλ−(0+)]DN(t)dt    +lim⁡N→∞∫0πgλ−(0+)DN(t)dt,    lim⁡N→∞∫0π[gλ+(t)−gλ+(0+)]DN(t)dt    +lim⁡N→∞∫0πgλ+(0+)DN(t)dt]  =[π2gλ−(0+),π2gλ+(0+)]=π2gt(0+).

**Theorem 41**

Let *f* ^*t*^ be any 2*π*-periodic continuous fuzzy-valued function and H-differentiable on [−*π*, *π*]. The Fourier series of fuzzy-valued function converges to

- *f* ^*t*^(*x*) for every value *x*, where *f* ^*t*^ ∈ *C*_*F*_[−*π*, *π*] for each *λ* ∈ [0,1],
- the arithmetic mean of the right-hand and left-hand limits *f* ^*t*^(*x*−) and *f* ^*t*^(*x*+) which are given in Definition 37, where the one-sided limits at each point of discontinuity exist.

**Proof**

(i) Firstly, continuity and the existence of one-sided H-derivatives are sufficient for convergence. Secondly, if *f* ^*t*^ ∈ *C*_*F*_[−*π*, *π*] at *x*, it follows that *f* ^*t*^(*x*+) = *f* ^*t*^(*x*) = *f* ^*t*^(*x*−), so the Fourier series of fuzzy-valued function converges to *f* ^*t*^(*x*) for all *x*, *t* ∈ [−*π*, *π*] and for each *λ* ∈ [0,1].

(ii) The continuity means that Fourier fuzzy coefficients *a*_*n*_ and *b*_*n*_ exist for all appropriate values of *n*, and the corresponding Fourier series for *f* ^*t*^ is given by (43). The *N*th partial level sum *S*_*N*_ of the series in (43) is

(74) ft(x)≅12π[(FH)∫−ππft(x)dx]λ ⊕⊕∑n=1N1π[(FH)∫−ππft(x)cos⁡⁡(ns−nx)dx]λ.

Since the first property of Dirichlet kernel *D*_*N*_(*s* − *x*) = (1/2) + ∑_*n*=1_^*N*^cos⁡⁡(*ns* − *nx*), using the partial level sum in (74), we get

(75) SNt(x)=1π[(FH)∫−ππft(x)DN(s−x)dx]λ=1π[∫−ππfλ−(t)DN(s−t)dt,      ∫−ππfλ+(t)DN(s−t)dt]

for *x*, *t* ∈ [−*π*, *π*] and *s* ∈ ℝ. By using 2*π*-periodicity of *f* ^*t*^ and the Dirichlet kernel in Lemma 38, we have

(76) SNt(x)=1π[∫t−πt+πfλ−(t)DN(s−t)dt,     ∫t−πt+πfλ+(t)DN(s−t)dt].

The integral in (76) splits into the two following integrals:

(77) SNt(x)=1π∫t−πt[fλ−(t)DN(s−t)dt,      fλ+(t)DN(s−t)dt] +1π∫tt+π[fλ−(t)DN(s−t)dt,         fλ+(t)DN(s−t)dt].

Each integral on the right-hand side can be simplified using Lemma 40, after making an appropriate change of variable. For the first integral, the change of variable will be *u* = −*t* + *s* so that

(78) ∫t−πtfλ±(t)DN(s−t)dt  =−∫π0fλ±(s−u)DN(u)du  =∫0πfλ±(s−u)DN(u)du.

Suppose that [*f*_*λ*_^−^(*s* − *u*), *f*_*λ*_^+^(*s* − *u*)] = [*f* ^*s*−*u*^(*t*_0_)]_*λ*_ = [*g* ^*u*^(*t*_0_)]_*λ*_ = [*g*_*λ*_^−^(*u*), *g*_*λ*_^+^(*u*)] for all *t*_0_ ∈ [0, *π*] in (78). Since the functions *g*_*λ*_^±^ are piecewise continuous on ]0, *π*[ and *g*_*R*_^*u*^(0) exists, to establish the existence of the right-hand H-derivative of *g* ^*u*^(*t*_0_) at *t*_0_ = 0, we have

(79) $$\begin{array}{c} {\left(g^{u}\right)}_{R}^{\prime }\left(0\right)=\underset{t\to 0+}{\lim}{\left[\frac{g^{u}(t)⊖g^{u}(0+)}{t⊖0+}\right]}_{\lambda }, \end{array}$$

where *g* ^*u*^(0+) = lim⁡_*t*→0+_[*g* ^*u*^(*t*)]_*λ*_ = lim⁡_*t*→0+_[*f* ^*s*−*u*^(*t*)]_*λ*_ = lim⁡_*s*→*u*_ *f* ^*t*^(*s* − *u*) = *f* ^*t*^(*x*−). Consequently, we derive that

(80) lim⁡N→∞∫t−πtfλ±(t)DN(s−t)dt  =lim⁡N→∞∫0πfλ±(s−u)DN(u)du  =lim⁡N→∞∫0πgλ±(u)DN(u)du  =π2gu(0+)=π2ft(x−).

The second integral on the right-hand side of (77) is analysed in a similar way. In this case, the change of variable is *u* = *t* − *s*. Suppose that if we take [*f*_*λ*_^−^(*s* + *u*), *f*_*λ*_^+^(*s* + *u*)] = [*g*_*λ*_^−^(*u*), *g*_*λ*_^+^(*u*)], then

(81) lim⁡N→∞∫tt+πfλ±(t)DN(t−s)dt  =lim⁡N→∞∫0πgλ±(u)DN(u)du  =π2gu(0+)=π2ft(x+).

By taking into account (80) and (81), and if we let *N* → *∞* in (77), then we have

(82) lim⁡N→∞1π[∫t−πt[fλ−(t)DN(s−t)dt,        fλ+(t)DN(s−t)dt]    +∫tt+π[fλ−(t)DN(s−t)dt,         fλ+(t)DN(s−t)dt]]  =12(ft(x−)+ft(x+)).

This completes the proof.

We assume that the above results hold with respect to 2*π*-periodic fuzzy-valued functions. The similar results can be obtained for a continuous H-differentiable periodic fuzzy-valued function of an arbitrary period *P* > 0.

## 5. Conclusion

As conventional hardware systems have been based on membership functions, a membership grade has been assigned to each element in the universe of discourse [21]. In this way, a wide variety of membership-function forms are being implemented and may reduce the number of conditional propositions for fuzzy inference to generate complex nonlinear surfaces, such as those used in fuzzy control and fuzzy modeling. More complex surfaces can be generated with a limited number of conditional propositions, with increasing types of membership-function forms. This is an advantage over approximating membership functions, especially with triangular or trapezoidal forms. Indeed, some useful results have been obtained by using level sets for defining series of fuzzy-valued functions like Fourier series. The potential applications of the obtained results include the generalization of sequences and series of fuzzy-valued functions.

One of the purposes of this work is to extend the classical analysis to the fuzzy level set analysis dealing with fuzzy-valued functions. Some of the analogies are demonstrated by theoretical examples between classical and level set calculus. Of course, several possible applications on Fourier series over real or complex field can be extended to the fuzzy number space. We should record from now on that the main results given in Section 4 of the present paper will be based on examining Fourier analysis of fuzzy-valued functions. Future work will be dedicated to find some applications on Fourier series of these functions.
